# Control of Genome Stability by EndoMS/NucS-Mediated Non-Canonical Mismatch Repair

**DOI:** 10.3390/cells10061314

**Published:** 2021-05-25

**Authors:** Esmeralda Cebrián-Sastre, Isabel Martín-Blecua, Sonia Gullón, Jesús Blázquez, Alfredo Castañeda-García

**Affiliations:** 1 Centro Nacional de Biotecnología, Department of Microbial Biotechnology, Consejo Superior de Investigaciones Científicas (CSIC), 28049 Madrid, Spain; esmeralda.cebrian@cnb.csic.es (E.C.-S.); imartin@cnb.csic.es (I.M.-B.); sgullon@cnb.csic.es (S.G.); 2 Clinical Unit of Infectious Diseases, Microbiology and Preventive Medicine, Instituto de Biomedicina de Sevilla (IBiS), 41013 Sevilla, Spain

**Keywords:** EndoMS/NucS, non-canonical mismatch repair, genome stability, hypermutation, antibiotic resistance, Actinobacteria, *Mycobacterium tuberculosis*

## Abstract

The DNA repair endonuclease EndoMS/NucS is highly conserved in Archaea and Actinobacteria. This enzyme is able to recognize and cleave dsDNA carrying a mismatched base pair, and its activity is enhanced by the interaction with the sliding clamp of the replisome. Today, EndoMS/NucS has been established as the key protein of a non-canonical mismatch repair (MMR) pathway, acting specifically in the repair of transitions and being essential for maintaining genome stability. Despite having some particularities, such as its lower activity on transversions and the inability to correct indels, EndoMS/NucS meets the main hallmarks of a MMR. Its absence leads to a hypermutator phenotype, a transition-biased mutational spectrum and an increase in homeologous recombination. Interestingly, polymorphic EndoMS/NucS variants with a possible effect in mutation rate have been detected in clinical isolates of the relevant actinobacterial pathogen *Mycobacterium tuberculosis*. Considering that MMR defects are often associated with the emergence of resistant bacteria, the existence of EndoMS/NucS-defective mutators could have an important role in the acquisition of antibiotic resistance in *M. tuberculosis*. Therefore, a further understanding of the EndoMS/NucS-mediated non-canonical MMR pathway may reveal new strategies to predict and fight drug resistance. This review is focused on the recent progress in NucS, with special emphasis on its effect on genome stability and evolvability in Actinobacteria.

*Thanks, Miro, for being always an inspiring and stimulating fresh air in the field of DNA repair and evolution.*

## 1. Introduction

Exogenous and endogenous factors produce damage in DNA that needs to be repaired in order to avoid detrimental effects on the cells, such as mutations and eventually, cell death. Therefore, the existence of efficient DNA repair pathways is essential to counteract DNA damage and maintain genome stability [1]. The main DNA repair pathways conserved from prokaryotes to eukaryotes include base excision repair (BER), nucleotide excision repair (NER), mismatch repair (MMR), homologous recombination (HR) and non-homologous end-joining (NHEJ) [2].

The MMR system recognizes mismatched bases, which mainly occur after replication as a result of the incorporation of non-complementary nucleotides leading to point mutations, or strand slippage forming small indels at repetitive sequences. Hence, the MMR activity enhances the DNA fidelity by 100- to 1000-fold [3]. This pathway also prevents recombination between non-identical sequences (homeologous recombination) by identifying DNA mismatches between recombination intermediates [4]. Additionally, the MMR system has a role in the DNA damage response, recognizing the mismatches generated by the chemical modification of bases due to DNA damaging agents [5]. To correct the mismatch, once it is detected, the MMR system excises and resynthesizes the fragment that contains the mismatched base in the newly synthetized strand [4].

The loss of this activity results in high spontaneous mutation rates, transition-biased mutational spectra and increased rates of homeologous recombination [2,6,7]. In addition, MMR defects cause resistance to the cytotoxic effects produced by certain DNA damaging agents [8]. Moreover, its inactivation increases the risk of cancer and the development of sporadic tumours in humans [9]. Therefore, the MMR repair has a pivotal role in maintaining genetic stability and its loss can have important biological consequences.

The bacterial canonical MMR systems require the MutS and MutL proteins to develop its repair activity. MutS recognizes mismatched base pairs and then MutL interacts with the MutS–DNA complex in an ATP-dependent manner. The MutS–MutL heteroduplex complex participates in the initiation of the MMR and activates endonuclease activity to nick the nascent strand (MutH in *Escherichia coli*, intrinsic MutL endonuclease activity in most prokaryotes) [10]. The MutS–MutL complex interacts with the bacterial sliding clamp (β-clamp), establishing a connection between DNA replication and the MMR system [11,12,13]. The β-clamp–MutS interaction directs MutS to the mismatched base [12,14], while in some organisms the interaction with MutL can stimulate its endonuclease activity and the mutants in the clamp-binding motif abolish the MMR activity [15]. After that, several specialized proteins, including helicases, exonucleases, the DNA polymerase III holoenzyme and DNA ligases, are involved in the excision repair of the incorrect base in the newly synthetized strand [4,16,17]. Strand recognition and selection are based on hemi-methylation in *E. coli* and closely related species, while the majority of bacteria may recognize a pre-existing nick probably produced by strand discontinuities [4,10].

MutS and MutL are highly conserved between prokaryotes and eukaryotes, but are absent in Actinobacteria and several groups of Archaea [18,19,20]. These organisms exhibit rates and spectra of spontaneous mutations comparable to the prokaryotes that harbour the canonical MMR, suggesting the existence of an alternative mechanism for the MMR [21,22,23]. Recently, the MMR-specific protein EndoMS/NucS has been described in Archaea and Actinobacteria, revealing the presence of a non-canonical MMR pathway in prokaryotes [20,24].

This review is focused on the recent findings on EndoMS/NucS (named, for the sake of simplicity, “NucS” from this point onwards) in Archaea and especially in Actinobacteria, a bacterial group including relevant members for industrial applications, such as *Corynebacterium glutamicum* and *Streptomyces sp.*, and important pathogens such as *Mycobacterium tuberculosis*. We have compiled biochemical, structural and genetic analyses focusing on actinobacterial NucS that provide relevant information about the characteristics of the non-canonical mycobacterial MMR and suggest its potential role in the emergence of antibiotic-resistant bacteria.

## 2. NucS, a Novel DNA Repair Protein in Prokaryotes: Characterization and Structure

NucS is a DNA repair endonuclease first identified as a novel archaeal nuclease specific for ssDNA in *Pyrococcus abyssi* [25]. It was originally isolated in a screening of *P. abyssi* DNA sliding clamp-binding proteins and characterized as a structure-specific DNA endonuclease [25,26]. More recently, it was identified searching for mismatch base-specific endonuclease activity in a *Pyrococcus furiosus* library and thus was renamed as EndoMS (mismatch-specific endonuclease), while its biochemical activity was fully characterized in the *Thermococcus kodakarensis* EndoMS homolog [24]. Although NucS was initially studied in Archaea, it has been shown that this DNA repair protein exhibits a wide distribution in those species lacking canonical MMR proteins, MutS and MutL, including Actinobacteria [20,24,27,28].

NucS is a highly conserved DNA repair protein in Actinobacteria and Archaea. Sequence alignments among representative members of the NucS family reflect that all of them retain a high degree of sequence identity and similarity [20,24]. Analysis of the sequence of NucS from *Mycobacterium smegmatis* and *M. tuberculosis*, actinobacterial members of this protein family, has unravelled important details related to the domain arrangement in this protein. It contains an N-terminal DNA-binding region, predicted to be the recognition site for the DNA substrate, followed by a C-terminal catalytic region responsible for the cleavage of the DNA, supporting its nuclease enzymatic activity [20].

NucS is folded in a two-domain structure with an N-terminal DNA-binding domain connected by a short linker to the C-terminal catalytic domain in Archaea [25,29]. Analysis of the archaeal NucS structure revealed that the enzyme acts as a dimer, with the two N-terminal binding domains in the middle attached by a large hydrophobic core, while the two C-terminal catalytic domains are arranged apart on both sides in an asymmetric display (C_1_ N_2_ N_1_ C_2_) [25,29]. Importantly, modelling of the enzyme structure in mycobacterial NucS supports that the folding of this endonuclease is also highly conserved in Actinobacteria [20].

The crystal structure of the archaeal NucS also showed that the N-terminal DNA- binding domain is folded in a unique half-closed β-barrel structure that comprises two layers of anti-parallel sheets (eight β-strands in total plus one additional α-helix). This region is also involved in enzyme dimerization, which is required for proper protein folding and stabilization. Structural comparisons reveal that the NucS N-terminal domain has a unique fold with certain similarities to ssDNA binding proteins [25]. The C-terminal catalytic domain contains an α/β fold built with a core of five-stranded central β-sheets and four flanking α-helices. This domain resembles a short endonuclease fold with an active site that conserves sequence motifs found in RecB-like nucleases [25,30].

The structure of the archaeal *T. kodakarensis* NucS–dsDNA complex reflected how NucS binds to its substrate [29,31]. The mismatched bases are flipped out from the dsDNA and arranged into a DNA binding site (recognition site) belonging to both N-terminal domains of the dimer, with a cluster of residues involved in the recognition and contact with the mismatched bases in the DNA. This attachment is prompted by a huge conformational change in the C-terminal domains, as revealed by structural comparisons between the unbound (apo) and bound form of *T. kodakarensis* NucS, required for proper recognition, positioning and cleavage of the DNA substrate [29].

## 3. NucS as a Mismatch Specific DNA Endonuclease: Activity and Specificity

The analysis of NucS enzymatic activity initially showed that NucS was a structure-specific endonuclease able to cut ssDNA/dsDNA junctions on branched substrates (flapped and sprayed DNAs) in archaeal *P. abyssi* [25,32,33]. However, further characterization in Actinobacteria (*C. glutamicum*) and Archaea (*P. furiosus* and *T. kodakaerensis*) revealed that NucS acts as an endonuclease able to recognize and cut a mismatched DNA substrate [24,34]. 

Actinobacterial NucS biochemical assays confirmed the specific cleavage of mismatched dsDNA substrates by the C-terminal NucS catalytic subunit in *C. glutamicum* [34]. While the purified wild-type NucS protein cleaved mismatched dsDNA, this activity was abolished in a catalytic mutant with inactive RecB-nuclease motifs in the C-terminal portion. This cleavage was powerfully enhanced by interaction with the sliding clamp of the replisome [34]. The requirement of this essential interaction for NucS activity, combined with different experimental conditions, could explain the absence of mismatch-specific cleavage in mycobacterial NucS *in vitro* [20]. 

**Figure 1:**
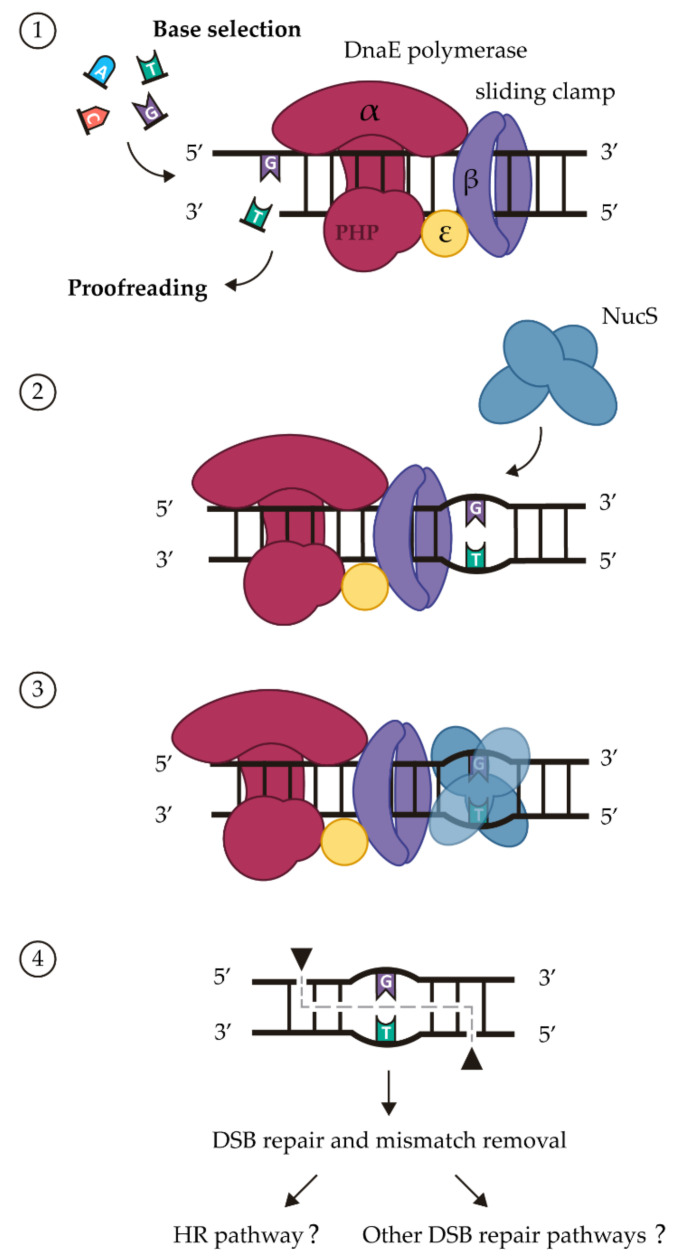
Model of action of the non-canonical MMR pathway in Actinobacteria. DnaE core polymerase (α subunit, red; ε subunit, yellow), sliding clamp (β subunits, purple) and NucS dimer (blue). (1) During replication DnaE polymerase performs base selection and, through its PHP domain, proofreading activity (3′–5′ exonuclease). In mycobacteria, ε subunit has no proofreading activity [36]. (2) The mismatches that escape these correction processes are the substrate of NucS. (3) NucS binds to the dsDNA containing a mismatch and its activity is stimulated by interaction with the sliding clamp. (4) NucS nicks both strands around the mismatch leaving a DSB. Finally, the DSB and the mismatch may be repaired through either HR pathway or other DSB repair mechanisms.

NucS cuts its substrate through double-stranded breaks (DSBs) to generate sticky ends comprising five overhanging nucleotides, with the mismatched base pair in the middle. The cleavage of each DNA strand occurs two nucleotides upstream (toward the 5´ end) from the mismatched pair, creating a DSB with 5´ protrusions (Figure 1) [24,29,34,35]. It has been proposed that NucS activity is closely related to type II restriction enzymes, sharing key signature motifs for enzymatic activity [29]. Furthermore, NucS is targeted to recognize and bind dsDNA containing a mismatched base pair but not properly paired dsDNA [20,24,34].

DNA-binding and cleavage assays indicate that the preferred substrates for NucS activity are G–T, G–G and T–T mismatches [24,34,35]. Weaker or no activity can be detected using other mismatches, especially noteworthy for the A–C mismatch. The narrower types of mismatches targeted by NucS seem to be specific and characteristic of this pathway in comparison with the broader substrates recognized by the MutS–MutL canonical MMR [37]. However, both of the systems share the same favourite target, the G–T mismatch, which leads to transition mutations in the DNA [37,38,39,40]. It has been proposed that this bias could be associated with the asymmetric accumulation of G–T mismatches (rather than A–C) as the predominant replication errors [34]. The structure of archaeal *T. kodakarensis* NucS supports that this endonuclease prefers G or T mismatched dsDNA as a substrate. The DNA-binding recognition site in the N-terminal portion provides stronger interactions when G or T, rather than A or C, occupy the base recognition site [29]. 

Finally, it has been shown that *Thermococcus gammatolerans* NucS can cleave uracil (U)- and hypoxanthine (I)-containing dsDNA, allowing to remove modified bases that arise from DNA deamination. The enzyme cleaves at the second phosphodiester on the 5′- site of the deaminated base, and at the third phosphodiester on the 5′-site of the opposite base of U or I, creating a double-strand break with a 4-nt 5′-overhang, which can be utilized by other DNA-repair proteins [41]. This adds another important function to NucS as part of an alternative pathway for the repair of deaminated bases.

## 4. NucS Pathway: Interaction with Protein Partners

As indicated, the cleavage activity of the endonuclease NucS was shown to be enhanced by the sliding clamp, both in Archaea and Bacteria [24,25,34,35,42,43]. In archaea the sliding clamp is the PCNA (proliferating cell nuclear antigen), which participates in the DNA processing during replication by tethering the catalytic subunit of the DNA polymerase and other proteins to DNA [44]. The interaction between the PCNA and NucS takes place through the PIP motif (PCNA-interacting peptide motif) [24,25,29,32,42,43], a short amino acid sequence that is found in many PCNA-binding proteins [45]. The PIP motif in *P. abyssi* NucS corresponds to 12 residues at the C-terminus of the protein [25], while *T. kodakarensis* NucS has a shorter PIP version with five C-terminal amino acids [24,29]. The structural model of the archaeal NucS–PCNA complex, supported by single-particle electron microscopy analysis, indicates that the NucS dimer binds through one PIP-box peptide to one of the three PIP-binding sites in the PCNA trimer to assemble into a stable protein complex [29].

The sequence of the PIP motif is also conserved in Actinobacteria in those proteins that bind to the sliding clamp, the β subunit of DNA polymerase III encoded by *dnaN* (the bacterial homolog of PCNA). This sequence of five amino acids residues, [E/K]–[L/Y]–[T/R]–L-F, is present in the C-terminal region of the protein NucS of many representative members of Actinobacteria, including *C. glutamicum*, *M. smegmatis*, *M. tuberculosis* and *S. coelicolor* [34,35]. The C-terminal NucS region was shown to be necessary for the interaction with the sliding clamp in *C. glutamicum* [35]. This interaction is essential for the activation of NucS, considerably increasing its cleavage efficiency *in vitro* [34,35]. The NucS–DnaN interaction is also required for the maintenance of a low spontaneous mutation rate *in vivo*, as shown when the binding of corynebacterial NucS to the sliding clamp was prevented by the deletion of the C-terminal residues of NucS [34,35]. 

NucS recognizes the mismatches that are left uncorrected by the proofreading activity of the polymerase, with the cleavage activity being promoted by the interaction with the sliding clamp. The subsequent mechanism for repairing the DSBs generated by NucS at the mismatch sites remains unknown, although the HR machinery of the cell is thought to be involved (Figure 1) [35]. It has been described that harbouring two or more chromosome copies, which occurs in some archaea and bacteria with non-canonical MMR [46,47], is an advantage for the use of HR to repair DSBs. However, it is not essential since non-polyploid organisms are able to utilize the HR pathway with this purpose [34].

Furthermore, in the genus *Thermococcus* of Archaea, *nucS* is co-transcribed with *radA*, the gene encoding the key protein for archaeal HR [24,25]. The bacterial homolog of the *radA* gene is *recA* [24], but in the case of Bacteria *nucS* and *recA* do not form part of the same operon. Whether RadA or RecA are involved in the correction of the DSBs produced by NucS-dependent activity has yet to be explored. Moreover, the following other putative *P. abyssi* NucS-interacting proteins, as well as PCNA, were found in a pull-down assay: Hef nuclease, small and large subunits of the replication factor C (the sliding clamp loader) and two subunits of topoisomerase VI [25]. More studies are needed to decipher the rest of the NucS partners involved in the repair pathway in Archaea and Actinobacteria.

## 5. NucS-Dependent Non-Canonical MMR: Biological Role

NucS has been established as the central core of an alternative non-canonical MMR. Despite being completely unrelated to canonical MMR proteins (MutS and MutL), it displays MMR activity *in vitro* and develops the biological role of a MMR pathway *in vivo*. The absence of NucS produces a hypermutator phenotype, a transition-biased mutational spectrum, and an increase in homeologous recombination in *M. smegmatis*, a model organism for Actinobacteria. The next subsections discuss, in depth, all the genetic data that support these MMR hallmarks found in NucS.

### 5.1. Hypermutation: NucS Is Required for Mutation Avoidance

The biological role of *nucS* was deciphered by the genetic screening of a *M. smegmatis* insertion library, searching for strong hypermutators. The inactivation of the target gene *nucS* led to a hypermutator phenotype in *M. smegmatis* [20]. The role of NucS as an anti-mutator component has been studied in several species, ranging from Actinobacteria to Archaea, through *nucS* deletion mutants and analysis of their mutational rates by fluctuation tests, or the estimation of mutant frequencies using different antibiotic markers [20,34,35,43,48].

By fluctuation assays, the inactivation of *nucS* leads to increases in the mutation rate of around 2 log in *M. smegmatis* (86- to 150-fold, depending on the antibiotic marker used for selection), but also in other actinobacterial species such as *S. coelicolor* (108- to 197-fold) and *C. glutamicum* (280-fold) [20,34]. In all cases, the basal mutation rates are restored when the Δ*nucS* strain is complemented with a copy of the wild-type *nucS* gene, confirming the absence of *nucS* as responsible for the hypermutator phenotype. Significantly similar results are observed in *mutS-* or *mutL*-deficient *E. coli* strains, with 100- to 1000-fold increases in mutation rate in the MMR deficient strains [6].

In Archaea, the deletion of the crenarchaeotal *nucS* in *Sulfolobus islandicus* causes an outstanding 1100-fold increase in mutant frequency [43]. This, together with the *in vitro* studies, supports the role of NucS as a key player in the non-canonical MMR pathway also in Archaea. Strikingly, no increase in mutation rate was detected in *nucS*-deficient *Sulfolobus acidocaldarius*, showing instead an increased sensitivity to a variety of helix-distorting DNA damage agents [48]. Further efforts need to be conducted to explore the biological role of the NucS-dependent DNA repair pathway in Archaea.

### 5.2. Mutational Bias: NucS Prevents the Accumulation of Transition Mutations

Transitions are the most frequent BPS (base pair substitution) mutations due to errors during DNA replication, and they are the preferred substrate of the MMR system [49]. Therefore, the transition-biased mutational spectrum represents a well-established hallmark of the canonical MMR system [6,50]. To characterize its mutational profile, the type of mutations conferring resistance to rifampicin have been analysed by sequencing the target gene in wild-type and Δ*nucS* strains in *M. smegmatis* and *C. glutamicum* [20,35]. In both actinobacterial species, the wild-type strains acquire different BPSs in *rpoB*, mainly transitions, but also a few transversions and even indels, while the Δ*nucS* strains present a distinctive accumulation of a huge number of transition mutations (A:T > G:C and G:C > A:T) [20,35,51]. A similar transition-biased profile is revealed in *M. smegmatis* Δ*nucS* by a reporter assay able to analyse the correction of each specific type of mutation [51].

**Figure 2:**
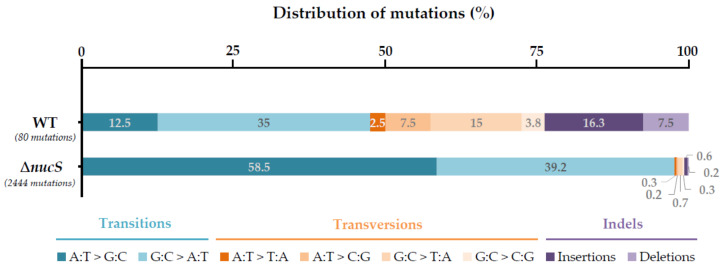
Distribution of each type of mutation in a MA assay of *M. smegmatis* wild-type and ∆*nucS* (according to [51]). Percentages are calculated with respect to the total number of mutations in the wild-type (80 mutations) and ∆*nucS* (2444 mutations) accumulated during 15,095 and 14,662 generations, respectively. Bars are divided in portions with different colours according to the type of mutation, as follows: transitions (blue tones), transversions (orange tones) and indels (purple tones).

The mutagenesis bias has also been thoroughly studied in *M. smegmatis* by mutation accumulation assays (MA), where the mutations are accumulated in a neutral manner [51]. In this study, the *nucS*-deficient strain exhibits an overall mutation rate ~31-fold higher than the wild-type. Focusing on the BPSs, the absence of *nucS* leads to a high increase in the number of transitions (~65-fold), with a shift in the mutational bias from G:C > A:T (the most common transition type in the wild-type strain) to A:T > G:C (Figure 2, Table 1) [51]. This change in the mutational bias suggests that NucS could participate in controlling the increase in the genomic G:C content, a function also attributed to the canonical MMR [6,51]. Regarding the number of transversions, it is very similar for the four types in the wild-type and ∆*nucS* strains, with no changes or only minor increases in the MMR-deficient strain. Lastly, no differences are observed in the number of short indels in presence or absence of *nucS* (Table 1). However, considering the higher number of mutations in the ∆*nucS* in respect to that of the wild-type, the proportion of transversions and indels is notably reduced in the *nucS*-deficient strain (Figure 2) [51].

A similar mutational spectrum has also been detected by MA assays in *C. glutamicum* (Table 1), where the absence of *nucS* results in an increase of ~74-fold in the number of transition mutations [34]. The *in vivo* mutational spectrum was also obtained in a *C. glutamicum* ∆*nucS*–*dnaE*(D647G) double mutant, a strain with a decreased DNA polymerase fidelity where the *nucS* gene was deleted [34]. A synergistic increase in the overall transition mutation rate can be detected in the double mutant, indicating that NucS cooperatively works with the DnaE polymerase to correct the replication errors across the genome [34]. An increase in the number of transitions was found as well in the archaeon *S. islandicus* ∆*nucS* through whole genome sequencing (WGS) [43].

In short, NucS is essential to maintain genome stability and integrity *in vivo*, acting specifically in the correction of transitions [51]. The higher efficiency in repairing transitions is shared by the MutS–MutL-based canonical MMR pathway [6,34,51,52,53,54,55]. However, it has some unique characteristics differing from the canonical MMR, such as its very low activity on transversions and its inability to correct short indels (Figure 2, Table 1) [6,34,51,52,53,54,55]. Moreover, the highly efficient exonuclease activity on both BPSs and indels of the PHP domain in the actinobacterial polymerase DnaE [36] could be responsible for the suppression of almost the totality of transversions and short indels that occur during the replication process, leaving for NucS the work of correcting the remaining BPSs, mainly transitions, that escape from this proofreading activity [51].

### 5.3. Recombination: NucS Interferes with Homeologous Recombination

The MMR contributes to controlling genome fidelity, not only by avoiding point mutations, but also by preventing homeologous recombination, establishing a barrier against interspecies recombination [7,56,57,58,59]. Thus, the inactivation of MMR genes not only results in hypermutable phenotypes, but also in increased homeologous (but not homologous) recombination rates [58]. In fact, the suppression of the canonical MMR allows interspecies recombination, as seen between *E. coli* and *Salmonella typhimurium* [60].

Recombination rates have been measured in *M. smegmatis* strains at different levels of sequence identity using specifically engineered tools [20]. When the sequences able to recombine were 100% identical, no differences in recombination rates were observed between the wild-type and the Δ*nucS* strain. However, in the absence of NucS, the recombination rate grows significantly between the non-identical sequences, reaching a 10-fold increase at 90% identity [20]. In addition, a previous report already reinforced the notion that there is a molecular barrier operating in mycobacteria to prevent intermolecular recombination [61]. The involvement of the MMR cascade in inhibiting homeologous recombination has been extensively verified as one of the distinctive features of the MMR system [62,63,64,65]. Hence, the fact that NucS modulates homeologous recombination is of high relevance to establish it as the core of the MMR system.

### 5.4. NucS Phylogeny, Filling the Gap in the Tree of Life

NucS and MutS–MutL MMR-dependent pathways share a common biological role, as reflected by the similar phenotypes exhibited by their null-derived strains. Considering their overlapping functions, it is not surprising that the taxonomic distribution of NucS and the canonical MMR proteins, MutS and MutL, strongly suggests that both MMR pathways are mutually exclusive. The tree of life can be separated into the following two different groups: species with the widespread canonical MMR MutS–MutL (lacking NucS) and species with the alternative non-canonical MMR NucS (but not MutS–MutL) [20,24]. Very few cases still remain with undetectable MutS–MutL or NucS, most of them with unknown mutation rates [20,24].

The NucS distribution pattern focused on the following two main groups of organisms: Actinobacteria and Archaea [20,24]. In Bacteria, the phylum Actinobacteria is vastly composed of NucS-encoding species, especially inside the central core of this group, the class Actinobacteria. In the case of Archaea, the presence of NucS is even wider, as it is the predominant pathway in the phylum Crenarchaeota, but it also reaches several important groups in the phylum Euryarchaeota [20,24]. Furthermore, the identified NucS-encoding species represent more than 10% of the bacterial species and 45% of all archaeal species with an available proteome. No eukaryotes or viruses have NucS [20]. The analysis of NucS domains in combination with its wider presence in multiple archaeal groups suggested an archaeal origin of this pathway [20,24]. In agreement with the clusters detected by phylogenetic analysis with the full protein and both domains (N-terminal and C-terminal) separately, the most parsimonious explanation suggests that NucS domains were first combined together in Archaea and then transferred to Actinobacteria by horizontal gene transfer (HGT) coupled with a MutS–MutL loss event in the last common ancestor of Actinobacteria [20].

The global distribution described for NucS and MutS–MutL reinforced the idea that both DNA pathways have evolved to correct the same type of lesion in the DNA. As there is no need to conserve two pathways with the same function, only one of them prevails, explaining this disperse and unique distribution. A few exceptions can be found for this rule, restricted to a few deinococci (Bacteria) and some halobacteria (Archaea). Interestingly, in *Halobacterium salinarium*, where both pathways coexist, *mutS* and *mutL* mutants were constructed and analysed, showing no effect in terms of mutant frequencies [66].

## 6. Hypermutability and Antibiotic Resistance Evolution in *M. tuberculosis*

In bacteria, alleles with increased mutation rates (mutators or hypermutators) are isolated in nature at a higher frequency than previously expected [67,68,69]. Because these alleles increase the possibility of favourable mutations, they can accelerate the evolutionary rate under some conditions. Mutators increase their frequency in the population by “hitch-hiking” with the favourable mutations that they have originated [70]. Thus, the appearance of a mutator allele would increase the chances of acquiring selective advantages, including antibiotic resistance, by mutational events.

Increases in the mutation rate are caused by decreased activity or loss-of-function mutations in bacterial genes that encode DNA replication, maintenance, and repair proteins [71]. Hypermutability in bacteria has been very often associated with defects in MMR components. Transient hypermutation may also occur by depletion of MMR activity as a response to antibiotic challenges [72]. Over the last two decades, strong evidence has been provided for a relevant role of mutators in bacterial infections, especially in chronic lung infections [73,74,75]. Hypermutability may have important effects, not only on antibiotic resistance, but also on the virulence, niche adaptation, persistence and transmissibility of pathogens [76,77,78]. The success of hypermutable alleles depends on the effect of mutations on the adaptation to a particular niche, the absence of HGT and the population size [79].

The discovery of the putative non-canonical MMR in mycobacteria has opened a whole set of new possibilities in the way we think about how *M. tuberculosis* protects its genome integrity and may respond to adaptive challenges. However, the study of DNA repair systems and their effect on mutation rates is still relatively unexplored in this deadly pathogen. Interestingly, *M. tuberculosis* appears to be genetically isolated, with a clonal population structure and little, if any, ongoing DNA transfer between strains [80,81]. It acquires antibiotic resistance exclusively by mutation and it presents variability in the mutation rates between strains [82,83]. The selection of hypermutable variants is, therefore, expected to be especially favoured in this pathogen. In fact, a correlation between high mutation rates and antibiotic resistance has been suggested for some *M. tuberculosis* lineages, such as the “Beijing family” [83,84,85], although this correlation was challenged by other studies [86].

The presence of missense SNPs (single-nucleotide polymorphisms) in *nucS* sequences from 1600 *M. tuberculosis* clinical isolates was studied by in silico analysis [20]. The effect of these polymorphic NucS variants on hypermutability was analysed by expressing them in a surrogate *nucS*-deficient *M. smegmatis* strain, previously shown to revert its hypermutable phenotype when complemented with a wild-type *M. tuberculosis nucS* gene [20]. Some of these missense SNPs (five alleles out of nine analysed) conferred significant increases in mutation rate over that of the wild-type *nucS* gene from *M. tuberculosis*. Although the number of missense SNPs characterized was very low, the proportion of putative mutators, ∼0.3%, (proportion of strains with SNPs in *nucS* out of all the 1600 studied strains) is much higher than expected according to the size of both the *M. tuberculosis* genome and *nucS* gene in the absence of selection. A search of *nucS* SNPs and indels, in a larger number of *M. tuberculosis* genomic sequences, has recently identified dozens of new NucS missense SNPs (A. Chiner-Oms and I. Comas, personal communication). Because NucS likely needs other partners to develop its activity, the number of naturally occurring mutator alleles detected in *M. tuberculosis* could increase proportionally. All these data suggest that *nucS*-associated hypermutability is under positive selection in clinical *M. tuberculosis* strains.

On the other hand, studies on antibiotic resistant *M. tuberculosis* isolates have shown that resistance is sometimes associated with a fitness cost and that this cost can be alleviated by compensatory evolution. For instance, WGS of rifampicin-resistant *M. tuberculosis* strains identified compensatory single mutations in RNA polymerase genes [87]. Hypermutation could also favour the acquisition of compensatory mutations [88], and this may benefit the success of *nucS*-derived drug resistant isolates.

The mutation rate of *M. tuberculosis* in clinical settings has been estimated to be orders of magnitude lower than in most bacterial pathogens (0.3–0.5 substitutions per genome per year) [89]. Nevertheless, despite this low mutation rate and the lack of ongoing HGT, antibiotic resistance mutations can arise quickly [90,91]. These apparent contradictory observations could be explained partially by the existence of *M. tuberculosis* stable or transient mutator strains.

## 7. Conclusions and Future Perspectives

NucS has been established as the key protein of the novel non-canonical MMR pathway, being essential for genomic stability and mutation avoidance. This enzyme is an endonuclease specific for mismatches and, even though it has a lesser role on transversions and indels, it shares the same preference for repairing transitions than the canonical MMR. As a result, the inactivation of non-canonical MMR leads to hypermutation, with strong increases in the rates of point mutations. In addition, NucS also seems to be involved in impeding homeologous recombination, avoiding interspecific recombination, in the same manner as MutS–MutL.

Hence, this non-canonical pathway fulfils all the main hallmarks of the canonical MMR. It is certain that NucS has a unique structure and a specific activity on the recognition and processing of mismatches by the generation of DSBs, reflecting its particular entity as an alternative MMR. Despite the differences regarding their components, the fact that both pathways play a similar biological role, together with the mentioned overlapping functions, points to a case of evolutionary convergence. As a result, the taxonomic distributions of these pathways are almost mutually exclusive.

Furthermore, the presence of SNPs in *nucS* from clinical *M. tuberculosis* isolates suggests the existence of hypermutable strains that are affected in NucS activity. Since *M. tuberculosis* acquires antibiotic resistance exclusively by mutation, the selection of hypermutable variants is particularly worrisome in this pathogen. Thus, understanding the participation of NucS in the control of *M. tuberculosis* genomic stability and the response to adaptative challenges could be highly relevant to tackle antibiotic resistance and virulence.

The discovery of NucS explains the genome stability and low mutation rates found in those organisms lacking canonical MMR, opening new possibilities in the field of DNA repair mechanisms. Still, to fully characterize the non-canonical MMR there are some questions that remain to be answered, relating to activity, pathway, mutational effect, regulation and expression, evolutionary consequences and clinical aspects (Table 2).

## Figures and Tables

**Table 1:** Increase in mutation rates of representative non-canonical and canonical MMR-deficient strains obtained from MA datasets.

	Fold Increase in MMR-Deficient Strains Mutation Rates ^1,2^			
	Non-Canonical MMR		Canonical MMR	
Type of Mutation	*M. smegmatis* ∆*nucS*	*C. glutamicum* ∆*nucS*	*E. coli* ∆*mutL*	*B. subtilis* ∆*mutS*
**Total BPSs**	**40.9**	**51.7**	**137**	**101.1**
**Transitions**	**64.7**	**73.4**	**238.2**	**130.7**
A:T > G:C	147.1	90.6	457.5	128.2
G:C > A:T	35.3	63.5	107.1	133.3
**Transversions**	**1.7**	**1.3**	**7.1**	**11.4**
A:T > T:A	3.6	0 ^3^	16.2	14.8
A:T > C:G	1	2.6	5.2	10.6
G:C > T:A	1.5	0.9	6.5	4.5
G:C > C:G	2.4	0	3.5	23.4
**Total indels**	**1**	**0**	**286.3**	**44.3**
Insertions	1.1	0	– ^4^	111.4
Deletions	0.7	0	–	22.9
**Overall**	**31.5**	**46.9**	**149.4**	**86.1**

^1^ MMR-deficient vs. wild-type strain mutation rates. ^2^ Data obtained from Castañeda-García et al., 2020 [51]. ^3^ Data with zero indicate that no mutations in the ∆*nucS* strain were detected for that type of change. ^4^ No data available.

**Table 2:** Outstanding topics to be addressed in the study of the non-canonical MMR pathway.

Future Perspectives	
**Activity**	- To decipher how the strand discrimination is performed to repair the newly synthesized strand. - To analyse the NucS ability to repair different DNA lesions, including those generated by chemical or physical damage.
**Pathway**	- To determine all the components of the non-canonical MMR, and the proteins that interact with NucS or belong to the repair pathway; - To know the mechanism of elimination and reconstitution of the mismatch-containing strand. - To decipher the mechanisms of processing the DSBs in DNA generated by NucS and evaluate its interplay with other DNA repair pathways, including recombination mechanisms.
**Mutation**	- To construct *nucS*-null mutants in different species, being especially relevant in some pathogenic mycobacteria, including *M. tuberculosis*, and to characterize the impact on hypermutability, fitness, evolvability, virulence and acquisition of antibiotic resistance. - To study the mutational response and susceptibility to different DNA damaging agents generated by *nucS* deficiency. - To explore the potential biotechnological applications based on the mutagenic effects generated by *nucS*-dependent genetic tools.
**Regulation** **and** **expression**	- To analyse *nucS* expression changes and regulation by intrinsic and/or extrinsic factors, and their effects on mutation rates and adaptation.
**Evolution** **and** **phylogeny**	- To compare non-canonical MMR pathways in Actinobacteria and Archaea, in terms of the similarities and differences. - To decipher the evolutionary origin of NucS-based MMR systems.
**Clinical**	- To characterize the emergence of drug-resistant strains under antibiotic pressure led by *nucS* deficiency. - To determine the existence of naturally occurring hypermutable variants, their relationship with *nucS* SNPs, and their effect on antibiotic resistance, virulence and adaptation to the environment/host in clinical strains.

## Data Availability

Not applicable.
